# Supplementary material for: Health Literacy Gaps Across Language Groups: A Population-Based Assessment in Alto Adige/South Tyrol, Italy
Source: Eur J Investig Health Psychol Educ. 2025 Aug 9;15(8):153. doi: 10.3390/ejihpe15080153 (PMC12386081; doi:10.3390/ejihpe15080153)
Supplement: Supplementary file 1 [file ejihpe-15-00153-s001.zip › ejihpe-3753663-supplementary.pdf]

Supplementary Material

Table S1. Mapping of HLS-EU-Q16 items to HLS-EU-Q47 source items and assigned conceptual domains.

HLS-EU-Q16 Item No.	Q16 Item Description (Abridged)	Corresponding HLS-EU-Q47 Item No.	Assigned Domain (From Q47)
Q1	Understand information on symptoms	1	Disease prevention
Q2	Understand what your doctor says	9	Health care
Q3	Know where to get professional help	13	Health care
Q4	Understand medication package leaflets	15	Health care
Q5	Judge when to see a doctor	16	Health care
Q6	Understand why vaccinations are needed	21	Disease prevention
Q7	Understand health risks	22	Disease prevention
Q8	Understand different treatment options	24	Health care
Q9	Find information on how to stay healthy	31	Health promotion
Q10	Decide if information on illness is reliable	32	Disease prevention
Q11	Get help to understand health information	33	Health promotion
Q12	Find information on mental well-being	38	Health promotion
Q13	Understand how to prevent mental health problems	39	Health promotion
Q14	Judge if information is applicable to you	40	Health promotion
Q15	Use information to make health decisions	41	Health promotion
Q16	Judge information from media sources	45	Health promotion

Table S2. Health literacy according to language subgroups other than German or Italian (weighted analysis).

Variable	Total (n = 280) [1]	Ladin (n = 79)	Other (n = 145)	More Than One (n = 56)	p-Value [2]	Effect Size [3]
HLS-EU-Q16 index (mean ± SD)	12.4 ± 2.9	12.3 ± 2.6	12.6 ± 3.3	12.8 ± 2.8	0.381	0.001 [4]
HLS-EU-Q16 level, % (n) [6]					< 0.001	0.246 [5]
Inadequate	9.4 (20)	9.0 (7)	7.6 (11)	3.6 (2)		
Problematic	23.7 (66)	37.2 (29)	12.4 (18)	33.9 (19)		
Sufficient	5.2 (126)	42.3 (33)	44.8 (65)	50.0 (28)		
Unknown/missing	24.0 (67)	11.5 (9)	35.2 (51)	12.5 (7)		

[1] Weighted proportions and weighted counts (rounded, shown in parentheses) are reported for the total and each subgroup. Groups include Ladin speakers, speakers of other single languages, and individuals reporting more than one primary language. Due to population weighting and variable-specific missing data, effective case numbers may differ from the unweighted sample totals shown in the header. Percentages are based on valid responses per variable. [2] p-values are based on Kruskal–Wallis tests for continuous variables and chi-squared tests of independence for categorical variables. [3] Effect sizes are reported as η^2 (eta-squared) for Kruskal–Wallis tests and Cramér's V for chi-squared tests. [4] η^2 = eta-squared. [5]Cramér's V. [6] HLS-EU-Q16 levels follow standard index thresholds: inadequate (0–8), problematic (9–12), sufficient (13–16). The "Unknown/missing" category includes cases with more than two missing responses on the 16-item scale.

Table S3. Intercorrelations between sociodemographic and health-related predictor variables (Spearman's rho) (*n* = 2090).

Predictor Variables	Gender (Vs. Male)	Age (Years)	Education Level (Vs. Middle School)	Non-Italian Citizenship (Vs. Italian)	Lives Alone (Vs. No)	Has Chronic Illness (Vs. No)	Works in Health/Social Sector (Vs. No)	Language: Italian (Vs. German)
Gender (vs. male)	–	0.016 (2219) n.s.	0.020 (2219) n.s.	–0.011 (2219) n.s.	0.063 (2219) ** [small]	0.031 (2219) n.s.	0.165 (2218) ** [small]	–0.003 (1936) n.s.
Age (years)		–	–0.392 (2219) ** [large]	0.154 (2219) ** [small]	0.129 (2219) ** [small]	0.403 (2219) ** [large]	–0.131 (2218) ** [small]	0.105 (1936) ** [small]
Education level (vs. middle school)			–	–0.109 (2219) ** [small]	–0.052 (2219) * [small]	–0.201 (2219) ** [small]	0.192 (2218) ** [small]	0.097 (1936) ** [small]
Non-Italian citizenship (vs. Italian)				–	0.008 (2219) n.s.	0.069 (2219) ** [small]	–0.007 (2218) n.s.	0.089 (1936) ** [small]
Lives alone (vs. no)					–	0.101 (2219) ** [small]	–0.016 (2218) n.s.	0.061 (1936) ** [small]
Has chronic illness (vs. no)						–	–0.038 (2218) n.s.	0.084 (1936) ** [small]
Works in health/social sector (vs. no)							–	–0.001 (1935) n.s.
Language: Italian (vs. German)								–

Notes: Spearman's rank correlation coefficients (ϱ) are shown for bivariate associations between sociodemographic and health-related predictor variables. Significance levels: $p < 0.05$, *; $p < 0.01$, **; n.s., non-significant ($p \geq 0.05$). The total weighted *n* reported in parentheses reflects effective sample size based on population weights (variable Weights). The unweighted sample size was 2,090 respondents. The value of 2,219 results from the sum of normalized weights used in weighted correlation estimation and may vary slightly across analyses due to rounding or case-wise missingness. Effect sizes in square brackets interpreted using Cohen's (1988) criteria.
